# Oral Healthcare Knowledge, Attitudes, Confidence and Learning Experiences Among Chinese Nursing Students: A Mixed-Methods Study

**DOI:** 10.1016/j.identj.2025.103994

**Published:** 2025-11-02

**Authors:** Xuancheng Chen, Linan Cheng, Yangyi Chen, Yuhuan Xie

**Affiliations:** a School of Nursing, Soochow University, Suzhou, China; b School of Nursing, Wenzhou Medical University, Wenzhou, China; c School of Nursing, Hangzhou Normal University, Hangzhou, China

**Keywords:** Oral health care, Knowledge, Attitude, Confidence, Nursing students

## Abstract

**Introduction and Aims:**

Oral diseases are widespread globally, yet nursing students often lack adequate education in oral healthcare. This study explores their knowledge, attitudes, confidence, and learning experiences to inform improvements in oral health education within nursing programs.

**Methods:**

A sequential mixed-methods design was conducted from April to July 2024 across three universities in Zhejiang and Jiangsu provinces, China. Quantitative data were collected via a structured questionnaire from 454 nursing students and analyzed using logistic regression. Subsequently, 21 students with high levels of oral healthcare attitudes and confidence were purposively selected for in-depth interviews. A phenomenological approach guided the qualitative phase, and data were used by thematic analysis .

**Results:**

Only 240 (52.86%) students demonstrated good levels of oral healthcare attitudes and confidence. Higher likelihood of positive attitudes and confidence was associated with being in the third-year, strong passion for nursing, good self-reported oral health and knowledge, and strong interest in working in oral healthcare. Qualitative analysis identified five themes: (1) learning pathways, (2) learning needs, (3) learning prospects, (4) influence on attitude, and (5) influence on confidence.

**Conclusions:**

Nursing students exhibited suboptimal attitude and confidence in oral healthcare, influenced by academic year, nursing passion, self-assessed oral health, knowledge, and interest in oral healthcare. Addressing learning barriers and integrating engaging, practice-oriented content may improve competence. Future research should assess these effect of such pedagogy on long-term clinical impact.

## Introduction

Oral diseases affect approximately 3.5 billion people worldwide, with dental caries being the most prevalent condition.^1^ Oral health encompasses the absence of pain, infections, periodontal diseases, tooth decay, tooth loss, and other disorders that may adversely affect overall physical, mental, and social well-being.^2^ It is crucial to general health and quality of life, as poor oral health can impair mastication,^3^ swallowing,^4^ nutrition,^5^^,^^6^ and contribute to systemic diseases such as cardiovascular disease,^7^ pneumonia,^8^ diabetes,^9^ and dementia.^7^^,^^10^ Consequently, oral health is increasingly recognized as a vital component of healthy aging.^11^

Oral healthcare plays a key role in preventing dental diseases and related complications,^12^ relying heavily on interdisciplinary collaboration among health professionals.^13^ Nurses are essential in integrating oral healthcare into routine nursing practice,^14^ and nursing students, as future nurses, require adequate oral healthcare education.^15^ However, studies indicate that nursing students often exhibit limited interest, inadequate attitudes, knowledge deficits, and low confidence in oral care,^16^, ^17^, ^18^, ^19^, ^20^ possibly due to insufficient educational exposure.^21^ Although interventions such as interprofessional education and curriculum enhancements have been proposed,^22^, ^23^, ^24^, ^25^ their effectiveness remains uncertain due to methodological challenges and resource constraints.

Most existing studies assessing nursing students' oral healthcare knowledge, attitudes, and confidence are cross-sectional and involve small or single-site samples, limiting comprehensive understanding.^17^^,^^26^, ^27^, ^28^ Given the importance of oral health and the urgent need to improve nursing education in this area, this study, guided by the Health Belief Mode (HBM),^29^ employs a mixed-methods approach. The quantitative phase evaluates nursing students' attitudes, confidence, and influencing factors, while the qualitative phase explores their learning experiences and how these shape attitudes and confidence. This research aims to provide a comprehensive understanding of nursing students' oral healthcare knowledge, attitudes, confidence, and related factors.

## Methods and materials

### Study design and participants

This study adopted a mixed-methods design, integrating both quantitative and qualitative approaches to comprehensively investigate nursing students' knowledge, attitudes, and confidence regarding oral healthcare. The HBM was used as the guiding theoretical framework. In the quantitative phase, perceived susceptibility, perceived severity, perceived benefits, perceived barriers, and self-efficacy were measured using items items measuring students' attitudes toward oral healthcare and their confidence in providing it, thereby examining how health beliefs influence their behavioral intentions. In the qualitative phase, the HBM informed the development of interview questions to explore nursing students' oral healthcare learning experiences (knowledge acquisition) and how these experiences shaped their attitudes and confidence, highlighting cues to action and contextual barriers.

The quantitative component employed a cross-sectional design and was reported in accordance with the STROBE (Strengthening the Reporting of Observational Studies in Epidemiology) guidelines.^30^ The qualitative component followed a descriptive phenomenological design guided by Giorgi's analytical framework ^31^ and findings were reported in line with the COREQ (Consolidated Criteria for Reporting Qualitative Studies) checklist.^32^

From April to July 2024, nursing students were recruited from three universities in Zhejiang and Jiangsu Provinces, China. Two of the universities were comprehensive institutions and one was a medical university, selected to ensure a diverse representation of nursing student populations. Inclusion criteria were: (1) full-time undergraduate nursing students, and (2) voluntary participation with informed consent. The exclusion criteria were: (1) students on campus due to leave of absence or suspension, and (2) those unwilling to participate.

## Measurements

### Structured questionnaire

A structured questionnaire was used in the quantitative phase, comprising three sections: sociodemographic information, oral healthcare-related items, and the Chinese version of the Attitude and Confidence with Oral Healthcare among Nursing Students (ACORN) scale.

Based on previous literature,^33^ sociodemographic variables included sex, academic year, self-rated GPA ranking, duration of clinical practicum/internship, and passion for nursing. Oral healthcare-related items consisted of self-rated oral health status, oral healthcare training experience, self-perceived knowledge and skills in oral healthcare, and willingness to engage in oral healthcare practice.

The ACORN scale was originally developed by Rojo et al.^21^ to assess nursing students' attitudes and confidence in oral healthcare. With the authors' permission, the scale was translated into Chinese and culturally adapted following Brislin's translation model.^34^ Validity and reliability testing were conducted among nursing students.

The Chinese version of the ACORN (C-ACORN) demonstrated good content validity, with an item-level content validity index (I-CVI) of 0.891 and a scale-level content validity index (S-CVI) of 0.906. Exploratory factor analysis (EFA) revealed four factors, explaining 72.626% of the cumulative variance. Confirmatory factor analysis (CFA) indicated a good model fit (χ²/df = 2.408, RMSEA = 0.077, NFI = 0.883, CFI = 0.928, TLI = 0.917, IFI = 0.928). The scale showed strong internal consistency (Cronbach's α = 0.938), and acceptable test-retest reliability (0.906) and split-half reliability (Spearman-Brown coefficient = 0.729).

The C-ACORN consists of 24 items across four subscales: (1) Attitude towards the role of nurses in oral health care, (2) Confidence in providing comprehensive oral health care, (3) Confidence in undertaking basic oral health assessment (e.g., recognition of healthy gums or tooth decay), and (4) Confidence in undertaking advanced oral health assessment (e.g., recognition of dental plaque or gum recession). Items are rated on a 7-point Likert scale, ranging from 1 (''strongly disagree'' or ''not confident at all'') to 7 (''strongly agree'' or ''very confident''), with total scores ranging from 24 to 168. Higher scores indicate more positive attitudes and higher confidence in oral healthcare, reflecting more effective implementation of oral health education strategies by nursing educators. Full details of the C-ACORN are provided in Appendix 1.

In this study, the Cronbach's α of the C-ACORN was 0.931, indicating excellent internal consistency. Following previous studies,^35^ students scoring equal to or above the mean were classified as having ''good attitudes and confidence'' in oral healthcare, while those scoring below the mean were considered to have ''poor attitudes and confidence. ''

### Outline of the semi-structured interview

A semi-structured interview guide was developed based on relevant literature and aligned with the study objectives.^36^, ^37^, ^38^ The guide included questions covering sources and preferences for acquiring oral health knowledge, personal learning experiences and impressions, suggestions for improving learning approaches, and the perceived significance of oral health knowledge in nursing practice. The guide was reviewed and revised by an expert panel consisting of a senior oral health nurse from a tertiary hospital, a nursing educator specializing in health literacy, and three postgraduate nursing students. To refine the content and estimate interview duration, a pilot test was conducted two weeks prior to the formal interviews with three nursing students meeting the inclusion and exclusion criteria. These students were selected through convenience sampling and were not included in the final study sample. Outline of the semi-structured interview can be found in Appendix 2.

### Data collection and quality control

Quantitative data were collected using a structured questionnaire administered through the secure online platform Wenjuanxing (https://www.wjx.cn/), which facilitated easy access and user-friendly participation. With permission from relevant institutional authorities, the survey link was distributed via DingTalk (a widely used instant messaging platform in China) to eligible nursing students using random sampling. Participants were informed about the anonymity of the study and their right to withdraw at any time without penalty. The online questionnaire included an introduction to the study, an informed consent section, and the main survey items. During the response process, researchers provided standardized explanations to address any questions raised by participants. After data collection, responses were reviewed, and invalid questionnaires were excluded based on patterned responses, logical inconsistencies, or completion times of less than 120 seconds, this cutoff was established based on previous research findings.

According to Kendall's rough estimation,^39^ the recommended sample size should be 10 to 15 times the number of questionnaire items. With 24 items and a 20% allowance for invalid responses, the required sample size was between 288 and 432. A total of 466 students were initially recruited, and all completed the questionnaire. After excluding 12 invalid responses, 454 valid questionnaires were analyzed, meeting the sample size requirements.

Qualitative data were collected through semi-structured interviews conducted via online video conferencing due to geographic constraints. Prior to participation, individuals were provided with detailed information regarding the study's purpose, confidentiality, estimated interview duration (approximately 30 minutes, based on pilot testing), and their right to withdraw at any time without consequence. Participants confirmed that their surrounding environment was safe, private, and had a stable internet connection. All interviews were conducted by a trained qualitative researcher in a quiet and private setting (e.g., an empty office or lab room) to ensure confidentiality. Open-ended questions were used to encourage free expression without leading the participants, and interviewers were experienced in qualitative research and had received formal training in qualitative methodology. Each interview lasted between 25 and 50 minutes and was audio-recorded, transcribed verbatim, and supplemented with field notes. Transcripts were returned to participants for member checking to ensure accuracy. All data were encrypted and securely stored on a password-protected computer accessible only to the research team.

**Fig. 1:**
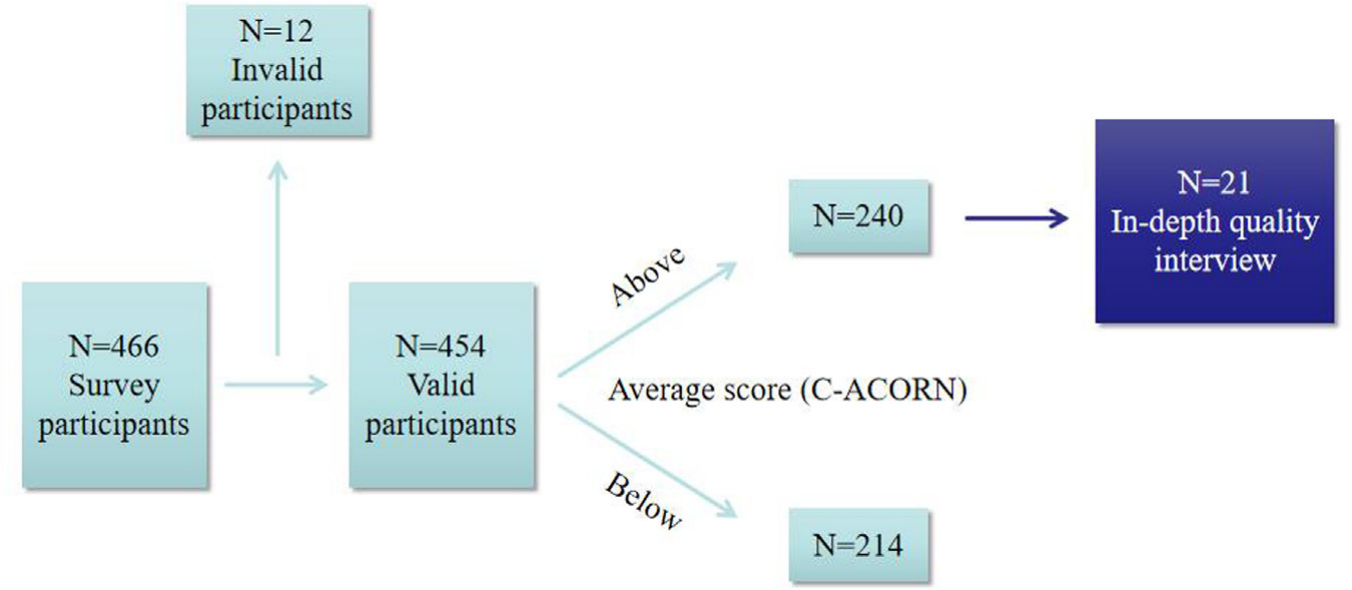
Flow chart of this study.

A purposive sampling strategy was adopted, with participants selected from those demonstrating higher levels of oral health attitudes and confidence. The number of interviews was determined based on data saturation, which was reached after 16 interviews. Five additional interviews were conducted to confirm no new themes emerged, resulting in a final sample of 21 interviews (Figure 1).

### Ethical considerations

This study was approved by the Ethics Review Committee of the School of Nursing, Hangzhou Normal University (2024008). All participates gave informed consent and voluntarily participated in the study. The study complied with the principles outlined in the Declaration of Helsinki.

### Data analyses

Quantitative data were analyzed using IBM SPSS software (version 25.0; IBM Corp., Armonk, NY, USA). Normality tests confirmed that all variables followed a normal distribution. Descriptive statistics (mean, standard deviation, median, and interquartile range) were used for continuous variables, and frequencies and percentages were reported for categorical variables. Independent sample t-tests and one-way analysis of variance (ANOVA) were performed to compare differences in oral healthcare attitudes and confidence among nursing undergraduates with different demographic characteristics. Binary logistic regression was used to identify factors influencing students' attitudes and confidence. A P-value of < .05 was considered statistically significant.

For the qualitative analysis, interview audio recordings were transcribed within 24 hours of completion. One researcher conducted the transcription and supplemented it with field notes, while another independently cross-checked the transcripts, audio files, and notes. The data were analyzed using Colaizzi's seven-step phenomenological method,^40^ by a nursing faculty member with extensive qualitative research experience. The steps included: (1) reading and familiarizing with the transcripts; (2) identifying significant statements; (3) formulating meanings from these statements; (4) clustering meanings into theme categories; (5) providing a detailed description of the phenomenon; (6) identifying the fundamental structure of the experience; and (7) returning the findings to participants for validation. An iterative coding process was used to develop subthemes and final themes. Throughout the analysis, the research team held multiple discussions to reach consensus on theme identification and refinement. To ensure clarity across language and cultural contexts, some quotations were slightly edited for readability.

## Quantitative study results

### Socio-demographic characteristics of participants

A total of 454 valid questionnaires were included in the final analysis, yielding an effective response rate of 97.42%. Among them, 81 (17.8%) were male and 373 (82.2%) were female. The mean age was 19.79 ± 0.57 years. First-year students accounted for 193 participants (42.5%). Regarding self-reported academic performance, 235 students (51.8%) reported a self-reported GPA ranking between the middle 30% and 60%. A majority (375, 82.6%) had less than three months of internship experience. In terms of passion for nursing, 259 (57.0%) reported having strong enthusiasm.

Self-reported oral health status was moderate for 204 students (44.9%), and 290 students (63.9%) had received oral healthcare training. A total of 299 students (65.9%) rated their oral healthcare knowledge as moderate, while 289 (63.7%) rated their oral healthcare skills as moderate. Additionally, 276 students (60.8%) reported having a strong passion for working in oral healthcare (Table 1).

**Table 1:** Univariate analysis of the attitude and confidence with oral healthcare among nursing students (n = 454).

Variables		Frequency	Percent %	t/F	P*-*Value
Sex	Male	81	17.8	-1.804	.074
	Female	373	82.2		
Year of study	First-year	193	42.5	3.827	.010*
	Second-year	149	32.8		
	Third-year	72	15.9		
	Fourth-year	40	8.8		
Self-reported GPA rank	Top 30༅	151	33.3	1.199	.302
	Middle 30༅-60༅	235	51.8		
	Bottom 40༅	68	15.0		
Internship duration	<3 months	375	82.6	0.967	.381
	3-6months	24	5.3		
	>6months	55	12.1		
Passion for nursing	Strong	259	57.0	0.113	.002⁎⁎
	Moderate	147	32.4		
	Weak	48	10.6		
Self-reported oral health	Good	158	34.8	13.366	<.001⁎⁎⁎
	Moderate	204	44.9		
	Poor	52	11.5		
Oral healthcare training	Yes	290	63.9	2.319	.021*
	No	164	36.1		
Self-reported oral healthcare knowledge	Good	106	23.3	12.784	<.001⁎⁎⁎
	Moderate	299	65.9		
	Poor	49	10.8		
Self-reported oral healthcare skills	Good	78	17.2	5.111	.006⁎⁎
	Moderate	289	63.7		
	Poor	87	19.2		
Passion for work in oral healthcare	Strong	276	60.8	6.597	.002⁎⁎
	Moderate	150	33.0		
	Weak	28	6.2		

⁎ *P*<.05.

⁎⁎ *P*<.01.

⁎⁎⁎ *P*<.001.

Students' attitudes and confidence in oral healthcare were significantly influenced by their academic year (*P = .*010), passion for nursing (*P = .*002), self-reported oral health (*P < .*001), prior oral healthcare training (*P = .*021), self-reported oral healthcare knowledge (*P < .*001), self-reported oral healthcare skills (*P = .*006), and passion for work in oral healthcare (*P = .*002) (Table 1). These findings offer further empirical validation of HBM. In accordance with the HBM framework, self-reported oral health status, which captures students' subjective experiences, appears to shape their perceived susceptibility to and severity of oral diseases. Additionally, self-reported knowledge and training are linked to perceptions of benefits and barriers. Moreover, the confidence assessed in this study aligns closely with the self-efficacy component of the HBM.

### The attitude and confidence regarding oral healthcare among nursing students

**Fig. 2:**
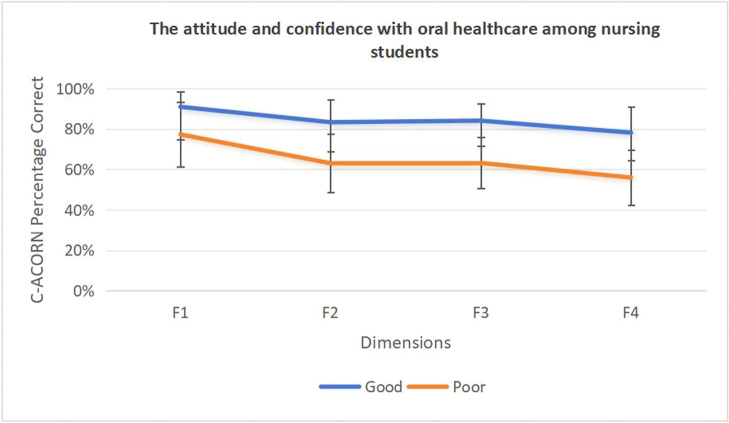
The attitude and confidence with oral healthcare among nursing students. F1, Attitude towards the role of nurses in oral health care;F2, Confidence in providing comprehensive oral health care;F3, Confidence in undertaking basic oral health assessment;F4, Confidence in undertaking advanced oral health assessment.

Among the 454 nursing students surveyed, 240 (52.86%) demonstrated good attitudes and confidence regarding oral healthcare, while 214 (47.14%) showed poor attitudes and confidence. Students with good attitudes and confidence reported higher scores across all four dimensions: attitude towards the role of nurses in oral health care (90.95% vs. 77.29%), confidence in providing comprehensive oral health care (83.32% vs. 63.04%), confidence in undertaking basic oral health assessment (84.10% vs. 63.19%), and confidence in undertaking advanced oral health assessment (78.17% vs. 55.98%). These comparisons were statistically supported by 95% confidence intervals and are presented in Figure 2.

### Factors associated with attitudes and confidence in oral healthcare among nursing students

Variables that showed statistical significance in the univariate analysis (Year of study, Passion for Nursing, Self-reported Oral Health, Oral Healthcare Training, Self-reported Oral Healthcare Knowledge, Self-reported Oral Healthcare Skills, and Passion for Work in Oral Healthcare) were included in a multivariate logistic regression model. The results revealed that all variables except Self-reported oral healthcare skills remained significantly associated with nursing students' attitudes and confidence toward oral healthcare (*P < .*05).

Compared with first-year students, third-year students were less likely to have negative attitudes and confidence (OR = 0.889, 95% CI = 0.491-1.608, *P = .*011). Students with moderate or weak passion for nursing were more likely to show lower attitudes and confidence than those with strong passion (OR = 2.098, 95% CI = 1.344-3.275, *P = .*005; OR = 1.283, 95% CI = 0.646-2.547, *P = .*001, respectively).

In terms of self-reported oral health, those who rated their oral health as moderate or weak were significantly more likely to report lower attitudes and confidence compared to those with good oral health (OR = 2.106, 95% CI = 1.317-3.370, *P < .*001; OR = 2.889, 95% CI = 1.625-5.138, *P = .*002).

Participants with poor self-reported oral healthcare knowledge were also more likely to have lower attitudes and confidence than those with good knowledge (OR = 2.774, 95% CI = 1.034-7.441, *P = .*043). Finally, those with weak passion for work in oral healthcare had significantly lower levels of attitude and confidence compared to those with strong passion (OR = 3.014, 95% CI = 1.123-8.093, *P = .*029). This suggests that motivational aspects, akin to cues to action in the HBM, also play a pivotal role in shaping students' oral healthcare attitudes and confidence as same as passion for nursing (Table 2).

**Table 2:** Multivariate analysis of factors associated with the attitude and confidence with oral healthcare among nursing students (n = 454).

Variable	B	SE	OR	95%CI		P*-*Value
				L	U	
Year of study (ref. First-year)						
Second-year	-0.621	0.245	0.537	0.332	0.869	.067
Third-year	-0.118	0.303	0.889	0.491	1.608	.011*
Fourth-year	0.024	0.373	1.024	0.493	2.127	.696
Passion for nursing (ref. Strong)						
Moderate	0.741	0.227	2.098	1.344	3.275	.005⁎⁎
Weak	0.249	0.350	1.283	0.646	2.547	.001⁎⁎
Self-reported oral health (ref. Good)						
Moderate	0.745	0.240	2.106	1.317	3.370	<.001⁎⁎⁎
Poor	1.061	0.294	2.889	1.625	5.138	.002⁎⁎
Oral healthcare training (ref. Yes)						
No	0.110	0.232	1.116	0.708	1.760	.635
Self-reported oral healthcare knowledge (ref. Good)						
Moderate	0.512	0.312	1.669	0.905	3.077	.101
Poor	1.020	0.503	2.774	1.034	7.441	.043*
Self-reported oral healthcare skills (ref. Good)						
Moderate	0.180	0.344	1.197	0.610	2.351	.601
Poor	0.332	0.431	1.394	0.598	3.248	.441
Passion for work in oral healthcare (ref. Strong)						
Moderate	0.107	0.226	1.112	0.715	1.732	.637
Weak	1.103	0.504	3.014	1.123	8.093	.029*

⁎ *P*<.05.

⁎⁎ *P*<.01.

⁎⁎⁎ *P*<.001.

## Qualitative study results

### Socio-demographic characteristics

A total of 21 participants were included in the qualitative study, with a mean age of 20.54 ± 0.62 years. Among them, 17 were female (81.0%). Most participants were third-year students. (n = 8, 38.1%). A majority (n = 16, 76.2%) reported a self-reported GPA ranking within the top 30%. Regarding the duration of internship, 12 participants (57.2%) had completed 3 to 6 months. Passion for nursing was reported as strong by 12 participants (57.1%).

Self-reported oral health was rated as moderate by 11 participants (52.4%). Most participants (n = 18, 85.7%) had not received oral healthcare training. 16 participants (76.2%) reported a moderate level of oral healthcare knowledge, and 13 (61.9%) reported moderate oral healthcare skills. In terms of passion for work in oral healthcare, 11 participants (52.4%) indicated strong passion (Table 3).

**Table 3:** Socio-demographic characteristics of qualitative study participants (n = 21).

Variables		Frequency	Percent %
Sex	Male	4	19.0
	Female	17	81.0
Year of Study	First-year	2	9.5
	Second-year	4	19.1
	Third-year	8	38.1
	Fourth-year	7	33.3
Self-reported GPA rank	Top 30༅	16	76.2
	Middle 30༅-60༅	5	23.8
	Bottom 40༅	0	0
Internship duration	3 months	2	9.5
	3-6months	12	57.2
	>6months	7	33.3
Passion for nursing	Strong	12	57.1
	Moderate	6	28.6
	Weak	3	14.3
Self-reported oral health	Good	5	23.8
	Moderate	11	52.4
	Poor	5	23.8
Oral healthcare training	Yes	3	14.3
	No	18	85.7
Self-reported oral healthcare knowledge	Good	3	14.3
	Moderate	16	76.2
	Poor	2	9.5
Self-reported oral healthcare skills	Good	4	19.0
	Moderate	13	61.9
	Poor	4	19.1
Passion for work in oral healthcare	Strong	11	52.4
	Moderate	8	38.1
	Weak	2	9.5

**Fig. 3:**
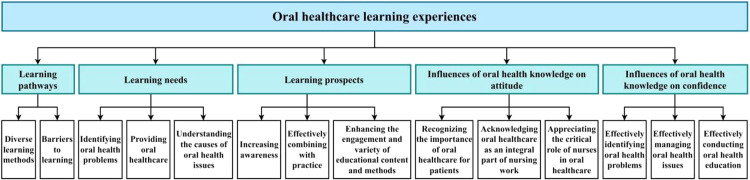
Themes and subthemes framework of oral healthcare knowledge learning experiences among nursing students.

Five main themes were identified from the in-depth interviews with participants. The first theme, '' Pathways to learn oral healthcare knowledge,'' included subthemes such as '' Diverse learning methods '' and '' Barriers to learning. '' The second theme, '' Needs for learning oral healthcare knowledge, '' encompassed subthemes like '' Identifying oral health problems,'' '' Providing oral healthcare, '' and '' Understanding the causes of oral health issues. '' The third theme, '' Prospects for learning oral healthcare knowledge, '' involved subthemes such as '' Increasing awareness, '' '' Enhancing the engagement and variety of educational content and methods, '' and '' Effectively combining with practice. '' The fourth theme, '' Influences of oral health knowledge on attitude, '' included subthemes like '' Recognizing the importance of oral healthcare for patients, '' '' Acknowledging oral healthcare as an integral part of nursing work, '' and '' Appreciating the critical role of nurses in oral healthcare. '' The fifth theme, '' Influences of oral health knowledge on confidence, '' involved subthemes such as '' Effectively identifying oral health problems, '' '' Effectively managing oral health issues, '' and '' Effectively conducting oral health education. '' Detailed descriptions are provided in Figure 3. The qualitative findings illustrate how learning experiences served as cues to action within the HBM framework. Gaining oral healthcare knowledge not only reduced perceived barriers but also enhanced self-efficacy, thereby strengthening both attitudes and confidence. Furthermore, students' reflections on the importance of oral healthcare highlight the role of knowledge acquisition in shaping perceived benefits and reinforcing their motivation to engage in oral health practice.

Theme 1 Pathways to learn oral healthcare knowledge

This theme reflects the diverse ways nursing students acquire oral healthcare knowledge as well as the multiple barriers they encounter during the learning process. Nursing students reported various learning pathways, ranging from everyday life experiences to formal school courses and clinical practice, indicating a wide diversity in learning approaches.

However, several barriers hinder effective learning. Most students primarily rely on the internet to obtain oral healthcare information but express skepticism regarding the professionalism and authority of online resources. Additionally, many students rarely engage in proactive learning of oral healthcare knowledge, partly due to limited learning opportunities. As Participant 4 stated, ''Apart from classroom learning, I seldom pay attention to oral healthcare knowledge. When I had my wisdom tooth extracted, I was worried about possible complications, so I searched extensively online for relevant information''.

Moreover, outdated or inadequate teaching equipment related to oral healthcare in schools also impedes effective learning. Although nursing students use diverse methods to learn oral healthcare, challenges such as difficulty verifying the credibility of online information, scarcity of learning opportunities, and obsolete educational tools remain significant obstacles to mastering oral healthcare knowledge.

This theme comprises two subthemes: ''Diverse ways'' and ''Barriers to learning oral healthcare knowledge.''

Diverse learning methods

Participants reported a diverse range of sources through which they acquired oral health knowledge, including formal coursework, clinical practice, dental consultations, family influence, online platforms, public service announcements, and educational lectures. Participant 1 remarked, *''In our Fundamentals of Nursing course, there was a dedicated session on oral healthcare, including practical training. During clinical rotations, my preceptor also introduced some basic oral health knowledge. Additionally, while walking outside, I often came across public health advertisements about dental care, especially around National Love Teeth Day.''* Participant 3 shared a multifaceted learning experience: *''I've primarily learned about oral healthcare through online videos. Another key moment was during a summer break in high school when I visited a dentist and received a comprehensive introduction to oral health. Later, in university, we studied a relevant chapter on oral care in our professional courses. In primary and secondary school, we occasionally had lectures on the topic. Finally, when I was a child, my family emphasized proper oral hygiene and brushing habits.''*

Barriers to learning

Participants conveyed several obstacles they encountered in the process of acquiring oral health knowledge. Although many relied primarily on digital media, concerns about the credibility and authority of online content were frequently raised. As Participant 6 noted, *''I mainly learn about oral health through short online videos, but I believe it is far better to rely on authoritative and official sources when studying this subject.''* Similarly, Participant 8 commented, *''I often come across oral health information while casually scrolling through the Xiaohongshu platform, but online content can be a mixture of truth and misinformation, so it really requires discernment.''* In addition, participants perceived limited opportunities for learning oral health knowledge and described the experience as largely passive. As Participant 1 observed, *''The amount of oral health content covered by our instructors is very minimal. Beyond formal coursework and clinical internships, we seldom take the initiative to engage with this subject.''* Participant 2 added, *''During hospital lectures, we occasionally learn about which types of mouthwash are suitable for specific patient needs or bacterial conditions. When attending such sessions, I sometimes search for supplemental information online, but lectures like these are relatively rare.''* Participants also expressed concern regarding the ineffectiveness of certain instructional tools used in oral health education. As Participant 2 pointed out, *'' Many of the oral care kits provided at school are opened only to reveal expired items, including swabs and cotton balls, which makes practice feel rather unrealistic.''* Echoing this sentiment, Participant 13 stated, *''We practice using mannequins and simulators. Sometimes, because these models lack subjective responses, it's easy to overlook the experiential aspect of real patient care.''*

Theme 2 Needs for learning oral healthcare knowledge

This theme reflects the multifaceted learning needs nursing students express regarding oral health. Many students articulated a desire to accurately identify both basic and complex oral health conditions, underscoring their need for a more comprehensive understanding of diagnostic indicators. Beyond the recognition of oral health problems, most students also emphasized the importance of mastering the procedures and protocols associated with providing oral healthcare, highlighting their aspiration to acquire operational competence in clinical contexts. In addition, some participants expressed a strong intellectual interest in the underlying mechanisms behind oral health issues. As Participant 7 remarked, *''When it comes to oral health problems, such as cavities or broken teeth, I'm curious about how these are related to the oral environment shaped by various microorganisms.''* Such expressions reveal not only the breadth of nursing students' curiosity, but also their current limitations in oral health knowledge and the pressing need for targeted educational interventions. This theme comprises three subcategories: *Identifying oral health problems, providing oral healthcare*, and *Understanding the causes of oral health issues*.

Identifying oral health problems

Participants expressed a clear need to develop the ability to identify oral health problems, aiming to ensure accurate assessment and timely intervention. As Participant 2 noted, *''It is not only important to recognize simple oral health issues, but also to identify more complex conditions.''* Echoing this view, Participant 7 remarked, *''I want to gain more detailed knowledge, such as how to determine whether or not a cavity is present.''* In addition to diagnosing clinical symptoms, participants also underscored the importance of recognizing behaviors detrimental to oral health. Participant 4 reflected, *''We often overlook certain harmful habits we engage in regularly. I want to understand what kinds of consequences they may lead to, and more importantly, why such consequences occur.''* Similarly, Participant 9 commented, *''I'd like to know what seemingly insignificant behaviors in daily life could negatively impact oral health*.''

Providing oral healthcare

Participants also expressed a strong desire to acquire practical knowledge related to the implementation of oral healthcare, particularly regarding the procedures and steps involved. As Participant 17 noted, *''I experience occasional pain in my wisdom teeth. When that happens, I usually take some anti-inflammatory medication for a few days and the pain subsides. So I'd like to learn how to properly manage wisdom tooth pain*.'' Participant 18 added, *''I want to understand how to respond appropriately to different dental conditions, for example, what is the correct way to relieve gum inflammation and swelling*?''

Understanding the causes of oral health issues

Participants demonstrated a strong intellectual curiosity regarding the underlying causes and mechanisms of various oral health issues, viewing such understanding as essential to deepening their knowledge and improving self-directed learning in oral healthcare. As Participant 3 remarked, *''It's not enough to simply know the basics of oral healthcare: we also need to understand the underlying principles and how these problems actually occur.''* Participant 11 elaborated further, *''For instance, among young people today, why does halitosis occur after staying up late or consuming spicy food? I'd like to understand the mechanism behind it.''*

Theme 3 Prospects for learning oral healthcare knowledge

This theme encapsulates nursing students' perspectives on effective strategies for learning oral health knowledge. Seeking guidance from professionals or utilizing specialized tools enables students to approach oral health issues from an expert standpoint, thereby facilitating a more nuanced and comprehensive understanding of oral healthcare concepts. Enriching the content and diversity of oral health education not only makes instruction more engaging but also fosters dynamic knowledge exchange between instructors and students, deepening learners' grasp of the material. Moreover, the integration of clinical practice emerges as a critical driver in promoting knowledge acquisition. Hands-on clinical experiences help solidify students' retention and comprehension of oral health principles. As Participant 17 described, *''During my internship, the clinical instructor used models for teaching and demonstrated how to assist patients. For instance, some patients who are unconscious are unable to brush their own teeth, so the instructor guided us step-by-step in providing oral care.''* Students' aspirations for oral health education stem not only from identified gaps in current knowledge but also from their authentic learning experiences. The pursuit of professional support, the enrichment of educational content and pedagogical methods, and the effective combination with clinical practice collectively provide valuable insights for enhancing oral health knowledge acquisition among nursing students. This theme is further subdivided into three categories: *Increasing awareness, Enhancing the engagement and variety of educational content and methods*, and *effectively combining with practice*.

Increasing awareness

Participants emphasized that consulting professionals on oral health matters serves as an effective means to acquire specialized knowledge, thereby enhancing their understanding and learning in this domain. As Participant 9 stated, *''I believe that seeking advice from relevant experts is a very good way to learn about oral healthcare. Their answers tend to be professional and easy to comprehend.''* Participant 14 further added, *''One can learn about oral health by consulting individuals with exemplary oral hygiene as well as professional clinicians.''* Furthermore, the majority of participants indicated that professional literature, educational videos, and books provide superior resources for learning oral health knowledge. Participant 8 remarked, *''I can refer to cutting-edge oral health research papers to keep my knowledge up to date.''* Participant 16 explained'', *There are some verified oral health practitioners online who share popular science videos; These can be valuable for learning how to properly maintain oral hygiene.''* Participant 21 suggested, *''Collaboration between clinicians and nurses to compile a specialized textbook would facilitate standardized learning of oral healthcare knowledge.''*

Enhancing the engagement and variety of educational content and methods

Participants highlighted the need to diversify both the content and formats of oral health education to facilitate deeper comprehension and retention of knowledge. Participant 15 remarked, *''I think incorporating specific oral healthcare case studies and presenting them in a more vivid and engaging manner would be beneficial, rather than relying heavily on textbook-style instruction, which feels rather rigid.''* Similarly, Participant 19 expressed, *''It would be helpful to include more entertaining popular science videos in the curriculum to enrich the teaching content; Otherwise, the learning experience can become quite monotonous.''*

Effectively combining with practice

Participants articulated that engaging in realistic or near-real clinical simulations and practical exercises allows them to intimately experience the authentic processes of oral healthcare. Such experiential learning bridges theoretical coursework with clinical practice, thereby enhancing comprehension and retention of oral health knowledge. As Participant 8 described, *''It would be valuable to select patients with typical oral diseases to conduct health education or to observe their treatment plans.''* Participant 13 added, *''I believe oral healthcare simulations could involve peer-to-peer practice or practice with close acquaintances, which would help us better appreciate the tactile and subjective aspects of care.''*

Theme 4 Influences of oral health knowledge on attitude

This theme reflects nursing students' perspectives on how oral health knowledge shapes their attitudes toward oral healthcare. Through their learning experiences, students progressively recognize the critical importance of oral care for patients' overall health and well-being, leading to a deep internal acceptance and acknowledgment of its significance. They also view oral healthcare as an indispensable component of nursing practice, with the prerequisite for effective implementation being a solid foundation of oral health knowledge. Moreover, students strongly affirm the pivotal role that nurses equipped with oral health expertise play in oral care, such as reducing infection risks and enhancing patients' quality of life. As Participant 10 articulated, *''Essential oral healthcare knowledge, in my view, can reduce the risk of infection among patients who are incapacitated or unconscious. At the same time, it can help those who are conscious and able to care for themselves to live more comfortably.''* Oral health knowledge not only reflects one's level of expertise but also significantly influences nursing students' attitudes toward oral healthcare. This theme is further subdivided into three categories: *Recognizing the importance of oral healthcare for patients, Acknowledging oral healthcare as an integral part of nursing work*, and *Appreciating the critical role of nurses in oral healthcare*.

Recognizing the importance of oral healthcare for patients

During their learning process, nursing students developed a profound appreciation for the importance of oral healthcare in patient well-being, which strongly shaped their positive attitudes toward its role in promoting health. Participant 19 expressed, *''I realize that oral care is crucial for patients. For example, if we do not provide oral care to an elderly lady, we might miss detecting a broken denture fragment, which could potentially cause choking and endanger her life.''* Participant 21 further added, *''Providing oral healthcare during hospitalization can reduce oral infections as well as respiratory infections. Additionally, maintaining a healthy oral environment supports better nutrition intake and overall recovery, ultimately benefiting the patient's condition.''*

Acknowledging oral healthcare as an integral part of nursing work

When discussing the significance of oral health knowledge in nursing practice, students overwhelmingly affirmed the view that oral healthcare constitutes an indispensable component of nursing duties. As Participant 1 stated, *''Oral healthcare is an essential element of nursing work. In this process, whether it be knowledge or skills, lacking either aspect means one cannot provide adequate oral care to patients.''* Participant 4 echoed this sentiment, noting, *''Oral healthcare is definitely part of nursing practice. If you plan to become a nurse but lack knowledge in this area, it reflects insufficient professional competence.''*

Appreciating the critical role of nurses in oral healthcare

Participants expressed strong endorsement of the pivotal role nurses play in oral healthcare. Participant 13 shared, *''Possessing solid oral health knowledge enables nurses to help patients reduce the risk of infections from other diseases. As the saying goes, 'disease enters through the mouth.' Effective oral care can prevent illnesses triggered by oral infections. Additionally, oral healthcare contributes to patients*' *physical comfort and psychological well-being.''* Participant 17 elaborated, *''Patients with prolonged hospital stays undoubtedly require oral care. Poor oral health can cause significant suffering. As nurses equipped with adequate oral health knowledge, we can promptly identify issues and alleviate patients' discomfort. I believe this is absolutely essential.*''

Theme 5 Influences of oral health knowledge on confidence

This theme reflects the role of oral health knowledge in shaping nursing students' confidence in delivering oral healthcare. Adequate mastery of oral health knowledge enables nurses to accurately identify oral health problems, laying the foundation for implementing appropriate and effective oral care interventions. Effective oral healthcare fundamentally depends on nurses' command of relevant knowledge. Health education is a critical component of oral healthcare, the successful delivery of which requires solid expertise in oral health. Participant 19 explained, *''When nurses possess the necessary oral health knowledge, I believe strengthening oral health education for patients becomes particularly important.''* The majority of nursing students concurred that nurses' proficiency in oral health knowledge influences their ability to effectively identify oral health issues, administer oral care, and conduct patient education. This underscores the crucial role of oral health knowledge in fostering confidence in oral healthcare delivery. This theme is further divided into three subcategories: *Effectively identifying oral health problems, effectively managing oral health issues*, and *effectively conducting oral health education*.

Effectively identifying oral health problems

Participants emphasized that possessing comprehensive oral health knowledge is instrumental in accurately identifying oral health issues, thereby laying a strong foundation for subsequent oral care interventions. As Participant 1 articulated, *''The foremost step is to thoroughly understand oral health knowledge. Only with such understanding can one accurately identify patients' oral health problems and determine the appropriate course of action.''* Participant 4 further elaborated, *''Equipped with essential oral health knowledge, nurses are better positioned to guide clinical practice and effectively recognize patients' oral health concerns.''*

Effectively managing oral health issues

Participants emphasized that possessing essential oral health knowledge empowers nurses to provide effective care for oral health issues. Participant 16 remarked, *''When nurses have the requisite oral health knowledge, they understand how to implement appropriate care measures to effectively alleviate patients' pain stemming from oral conditions and reduce unpleasant oral odors.''* Participant 21 further explained, *''If nurses are knowledgeable about oral healthcare, they can intervene in patients' oral health, thereby delivering effective management*.''

Effectively conducting oral health education

Participants articulated that a solid foundation of oral health knowledge enables nurses to provide effective oral health education to patients. As Participant 10 shared, *''I believe that nurses in clinical settings can utilize their oral health knowledge during their spare time to educate patients, especially since many of these patients are hospitalized.''* Participant 12 added, *''Nurses who possess comprehensive oral health knowledge are more effective in delivering oral health education. Moreover, patients tend to place greater trust in professionals who are well-informed in this area.''*

## Discussion

This mixed-methods study explored nursing students' experiences of learning oral healthcare knowledge, their attitudes, and confidence levels. Both quantitative and qualitative findings not only demonstrated differences in C-ACORN scale scores and predictors between students with good and poor attitudes and confidence, but also provided a comprehensive view of their learning experiences across knowledge acquisition pathways, learning demands, future prospects, and the shaping of attitudes and confidence. By situating these findings within the HBM, the study elucidates how perceived benefits, barriers, cues to action, and self-efficacy collectively influenced attitudes toward oral healthcare and their confidence in performing related practices. This integration underscores the utility of HBM as a framework for explicating the mechanisms through which learning experiences shape behavioral intentions.

Quantitatively, 52.86% of nursing students exhibited good oral healthcare attitudes and confidence, consistent with baseline data from an Australian oral health intervention study and other cross-sectional surveys.^22^^,^^26^ This suggests that there remains room for improvement in students' attitudes and confidence toward oral healthcare. One possible reason is that oral healthcare education in Chinese nursing curricula is still in development, with limited content in undergraduate courses.^21^^,^^33^^,^^41^ Nursing educators often face challenges such as limited teaching resources, heavy curricular burdens, and lack of clinical practice opportunities in oral health care, which may hinder effective theoretical and practical training, leading to reduced student confidence and interest. In HBM terms, these institutional constraints represent significant perceived barriers, which can impede the development of positive attitudes and adequate self-efficacy. These findings imply an urgent need to strengthen oral healthcare education by enriching curriculum content, improving teaching resources, and increasing clinical practice opportunities to better prepare nursing students for effective oral healthcare delivery.

In this study, among the subscale scores of the C-ACORN, the dimension ''Confidence in undertaking advanced oral health assessment'' had the lowest percentage scores in both the good and poor attitude and confidence groups. Moreover, the difference in scores between these two groups on this dimension was significantly greater than that of other dimensions, indicating that nursing students' attitudes and confidence toward oral healthcare are primarily constrained by their lack of confidence in recognizing and assessing complex oral health issues. This may be because this dimension reflects students' ability to identify complex or rare oral health problems, and the current deficiency in theoretical and practical education related to oral healthcare^42^ results in insufficient opportunities for students to acquire relevant knowledge and clinical skills during their training. Consequently, their confidence in handling complex cases is especially inadequate. Therefore, strengthening undergraduate nursing education in advanced oral health assessment through enhanced theoretical instruction and clinical practice, with particular emphasis on the identification of complex and uncommon oral health problems, may represent a critical yet often overlooked strategy to improve nursing students' attitudes and confidence in oral healthcare.

This study found that compared to first-year, third-year nursing students had a significantly lower likelihood of exhibiting poor attitudes and confidence toward oral healthcare (OR = 0.889). This difference may be attributed to variations in early clinical exposure.^43^ In China, nursing students typically complete approximately one month of clinical internship before their third academic year. Adequate early clinical exposure helps students deepen their understanding of oral healthcare knowledge taught by educators and may stimulate their interest in pursuing this field in the future, thereby enhancing their attitudes and confidence. This finding is further supported by qualitative data, where some participants reported gradual improvements in their attitudes and confidence through learning oral healthcare during clinical practice. Additionally, early integration of oral healthcare content within coursework and practical training can provide foundational knowledge and skills that enhance students' understanding and engagement.

Participants with weak or moderate passion for nursing were 2.547 and 3.275 times more likely, respectively, to report poor attitudes and confidence toward oral healthcare compared to those with strong passion. A possible explanation is that their intrinsic motivation for learning may be relatively low,^44^ making them less willing or less able to actively engage in learning and mastering oral healthcare knowledge and skills, which in turn negatively influences their attitudes and confidence. This finding is supported by the qualitative analysis, where some participants described their learning experience in oral healthcare as largely passive, with limited initiative and engagement.

Self-rated oral health status also played an important role in shaping nursing students' attitudes and confidence. Compared to those who rated their oral health as good, participants who reported moderate or poor oral health were 2.106 and 2.889 times more likely, respectively, to have poor attitudes and confidence toward oral healthcare. Interestingly, this finding contradicts the results of a previous cross-sectional study conducted in China, which found a negative association between nursing students' oral health literacy and their attitudes and confidence in oral healthcare.^33^ This discrepancy can be explained through the lens of self-efficacy theory.^45^ Nursing students who perceive their own oral health as poor may experience lower self-efficacy, leading to diminished confidence in providing oral healthcare or delivering oral health education to patients. This explanation is also supported by a cross-sectional study from Tanzania, which found a similar relationship between nursing students' oral health knowledge, beliefs, and practices.^28^ Although this association was not explicitly mentioned in the qualitative interviews, many participants described being highly motivated to learn oral healthcare knowledge during their own dental treatment experiences, in order to facilitate treatment or reduce complications. This process may have indirectly influenced their attitudes and confidence toward oral healthcare. These personal experiences may have functioned as cues to action, motivating further engagement in oral healthcare learning.

Self-assessed oral healthcare knowledge emerged as a significant factor influencing attitudes and confidence toward oral healthcare. Nursing students who rated their knowledge as good were more likely to demonstrate higher levels of oral healthcare attitudes and confidence. This finding is consistent with studies conducted in Japan ^27^ and the United States,^46^ which similarly indicated that enhancing oral healthcare knowledge can effectively improve related attitudes and confidence. This observation aligns well with the qualitative themes ''Influences of oral health knowledge on attitude'' and ''Influences of oral health knowledge on confidence,'' which describe in detail how knowledge acquisition contributes to the development of more positive attitudes and greater confidence among nursing students regarding oral healthcare.

Passion for work in oral healthcare was identified as a strong positive predictor of both attitude and confidence toward oral health practices. Participants who expressed a willingness to engage in oral healthcare-related work in the future generally reported higher levels of attitude and confidence, consistent with findings from a cross-sectional study conducted in Japan.^17^ Those who were interested in oral healthcare and intended to pursue it professionally tended to approach the learning of oral health knowledge and skills with a more proactive attitude. They actively sought learning opportunities and adopted various effective strategies to overcome barriers to learning. These behaviors ultimately contributed to the development of more positive attitudes and greater confidence in delivering oral healthcare services to patients. This finding also aligns with the qualitative theme *''Prospects for learning oral healthcare knowledge*,'' in which participants described or proposed strategies to cope with challenges encountered in the process of learning oral health knowledge.

Notably, the qualitative component of this study highlighted the complex and detailed experiences of learning oral healthcare knowledge that were not fully captured by the quantitative survey. Under the theme ''Ways to learn oral healthcare knowledge,'' participants described a wide range of approaches to acquiring knowledge, including online resources, formal coursework, clinical practice, and personal experiences during dental visits. These diverse learning pathways are consistent with contemporary nursing students' learning habits and preferences,^38^^,^^47^^,^^48^ underscoring the multifaceted nature of their educational experiences in oral health.

However, despite access to various learning pathways, several significant barriers hinder nursing students from effectively acquiring oral healthcare knowledge. These include concerns about the credibility of online resources, limited learning opportunities, low learning motivation, and outdated dental equipment in educational settings. These challenges resonate with findings from recent studies in the Netherlands, where barriers such as administrative burden, unclear quality definitions, and limited availability of resources were identified as hindering effective oral health practices across multiple stakeholder levels.^49^ While online platforms play a crucial role in supporting self-directed learning among nursing students, the reliability and accuracy of online health information remain questionable, posing a major challenge to obtaining trustworthy knowledge. Additionally, students' lack of initiative, coupled with insufficient institutional and clinical opportunities and outdated teaching infrastructure, may be attributed to the demanding nature of nursing curricula and clinical workloads,^50^^,^^51^ as well as the marginal emphasis placed on oral health education in assessments.^52^ Furthermore, these qualitative findings elucidate the underlying factors contributing to the comparatively low levels of oral healthcare-related attitudes and confidence identified in the quantitative phase. Consistent with observations from the Dutch context, our findings underscore the necessity of collaborative efforts among educators, institutions, and stakeholders to address systemic barriers and improve the practical integration of oral healthcare education. ^53^

The qualitative findings further revealed that participants had multilayered learning needs related to oral healthcare knowledge, including identifying oral health problems and unhealthy behaviors, implementing oral healthcare practices, and understanding the underlying causes of oral health issues. These diverse learning demands reflect a strong interest among nursing students in oral healthcare, which is consistent with findings from a Japanese study.^54^ This interest is also supported by the quantitative data, where 93.8% of participants expressed a willingness to engage in oral healthcare work. However, the quantitative data did not capture the specific content areas toward which this interest was directed, highlighting a gap between students' general enthusiasm for oral healthcare and the detailed, content-specific learning needs that support it.

The theme ''*Prospects for learning oral healthcare knowledge*'' emerged as a key aspect in understanding students' learning experiences. It reflects nursing students' perspectives and insights on effective strategies for acquiring oral healthcare knowledge. Participants described several approaches to overcoming learning barriers, including seeking professional support, enriching the appeal of educational content and formats, and integrating learning with practical experience. These strategies align with current trends in optimizing oral health education.^22^^,^^46^^,^^55^ While these approaches were not identified in the quantitative analysis, the qualitative findings underscore students' wisdom and innovative thinking, as their suggestions were grounded in personal experiences, formal education, and clinical practice.

The qualitative findings revealed that nursing students generally perceived oral health knowledge as having a significant impact on their attitudes and confidence. This insight complements the quantitative data by uncovering the complex mechanisms through which oral health knowledge influences students' attitudes and confidence, thus providing a more comprehensive understanding of their learning process and experiences. Within the HBM framework, this reflects the dual role of knowledge in reinforcing perceived benefits and reducing barriers, while simultaneously strengthening self-efficacy. Through their learning experience, students developed a deeper awareness of the importance of oral healthcare in both patient well-being and nursing practice, and they increasingly recognized the critical role of nurses in promoting oral health. Participants further noted that possessing adequate oral health knowledge enables nurses to identify oral problems, implement effective care strategies, and deliver health education. These findings are consistent with a mixed-methods study conducted in Australia^22^, which also underscored the significant influence of oral health knowledge on nursing students' attitudes and confidence levels.

## Conclusion

This mixed-methods study highlights the inadequacies in nursing students' attitudes and confidence toward oral healthcare and identifies several key influencing factors. Academic year, passion for nursing, self-reported oral health status, self-reported oral healthcare knowledge, and enthusiasm for working in the field of oral healthcare emerged as significant predictors of improved attitudes and confidence.

The qualitative findings further revealed that, although nursing students accessed a variety of oral health learning resources, including online platforms, coursework, clinical practice, and dental visits, the reliability of online information, limited learning opportunities, low intrinsic motivation, and outdated teaching and clinical equipment hindered their effective learning. Additionally, students articulated their coping strategies, learning needs, and recognized the importance of oral health knowledge in fostering positive attitudes and self-confidence.

These findings carry several practical implications. Nursing educators and administrators should prioritize updating teaching facilities and expanding opportunities for clinical practice to address the issue of limited resources. In curriculum design, emphasis should be placed on the identification of oral health problems, oral care practices, and patient health education skills. Incorporating engaging and hands-on teaching strategies may further stimulate students' motivation to learn. To enhance intrinsic motivation, strategies such as sharing real cases and underscoring the role of oral healthcare in overall patient well-being can be employed to strengthen students' passion for nursing and oral health work. Academic year-specific support measures should also be developed to address students' varying levels of competence. Furthermore, it is essential to assess disparities in oral healthcare capabilities among students and offer targeted, high-quality educational resources to reduce knowledge gaps.

Promoting self-awareness of personal oral health and establishing sustainable support systems, such as mentorship programs, can provide students with accessible and continuous professional guidance; Integrating oral healthcare education into undergraduate health curricula is crucial to ensure consistent learning goals and adequate resource allocation. Future research should explore the long-term impact of these integrated educational strategies on nursing students' attitudes, confidence, and clinical competence in oral healthcare practice.

## Limitations

This study employed a mixed-methods design to explore nursing students' experiences in learning oral healthcare knowledge, as well as their attitudes and confidence toward oral healthcare. By combining quantitative and qualitative approaches, the study provided insights into the levels of attitudes and confidence among nursing students and the complex mechanisms through which oral healthcare knowledge influences these factors. However, several limitations should be noted. First, the participants were recruited from three universities in Zhejiang and Jiangsu provinces, which may represent students from relatively economically developed regions. Future research should expand the geographic scope and increase sample size to further validate the external validity of the results. Second, in the qualitative phase, participants were purposively selected based on relatively high attitudes and confidence toward oral healthcare. While this approach allowed in-depth exploration of positive learning experiences, it may have underrepresented students with lower attitudes or confidence, who might experience different barriers and learning needs. Future qualitative studies could include participants with a broader range of attitudes and confidence levels to provide a more comprehensive understanding of challenges in oral healthcare learning. Third, this study relied solely on self-reported measures of attitudes and confidence. Such subjective assessments may be influenced by personal perception, social desirability, or recall bias, which could affect the precision and validity of the findings. Moreover, in China, validated and reliable tools for objectively assessing nursing students' oral healthcare knowledge are lacking. Future studies could incorporate standardized knowledge tests, case-based assessments, or practical skill evaluations alongside self-reported questionnaires and qualitative methods to provide a more comprehensive and accurate evaluation. Finally, as a cross-sectional study, causal inferences cannot be drawn. Longitudinal studies are needed to further investigate factors influencing nursing students' oral healthcare knowledge, attitudes, and confidence, especially across different curriculum stages and clinical experiences.

## Author contributions

Xuancheng Chen contributed to the study design, data collection, performed the statistical analysis, and drafted the initial manuscript. Linan Cheng contributed to the conceptualization of the research question, study design, interpretation of the results, supervision, as well as writing and critical revision of the manuscript. Yangyi Chen and Yuhuan Xie assisted in data collection. All authors reviewed and approved the final version of the manuscript.

## Data availability statement

The datasets generated during and/or analyzed during the current study are available from the corresponding author upon reasonable request.

## Funding

This research did not receive any specific grant from funding agencies in the public, commercial or not-for-profit sectors.

## Ethics statement

This study was approved by the Ethics Review Committee of the School of Nursing, Hangzhou Normal University (2024008). All participants gave informed consent and voluntarily participated in the study. The study complied with the principles outlined in the Declaration of Helsinki.

## Conflict of interest

The authors declare that they have no competing interests.

## Appendix Supplementary materials

Image, application 1

Image, application 2
