# Supplementary material for: Oral Healthcare Knowledge, Attitudes, Confidence and Learning Experiences Among Chinese Nursing Students: A Mixed-Methods Study
Source: Int Dent J. 2025 Nov 2;76(1):103994. doi: 10.1016/j.identj.2025.103994 (PMC12603743; doi:10.1016/j.identj.2025.103994)
Supplement: Supplementary file 1 [file mmc1.docx]

**The Chinese version of the Attitude and Confidence with Oral Healthcare among Nursing Students (C-ACORN)**

| **Dimensions and items** |
| --- |
| **Attitude towards the role of nurses in oral health care** |
| Q1. Nurses have the responsibility to ensure patients have good oral hygiene |
| Q2. Oral care provided to patients is as important as pressure sore care |
| Q3. It is important for nurses to refer patients in need of dental care |
| Q4. Nurses are very capable of discovering oral health problems in the early stage |
| Q5. Patients with dysphagia always need oral care |
| Q6. Reminding patients to participate in oral care is the responsibility of nurses |
| Q7. Nurses should have the skills to conduct oral health assessments |
| Q8. It is important to ensure that patients of all ages receive adequate oral care |
| **Confidence in providing comprehensive oral health care** |
| Q9. Discuss oral health with patients |
| Q10. Discuss the relationship between oral health and health status with patients |
| Q11. Provide comprehensive oral care for patients who are conscious but unable to get out of bed |
| Q12. Referring patients to dental services based on their oral health assessment results |
| **Confidence in undertaking basic oral health assessment** |
| Q13. Healthy Gums |
| Q14. Healthy teeth |
| Q15. Tooth decay |
| Q16. Pain in the mouth |
| Q17. Lips ulcer |
| Q18. Food residue |
| **Confidence in undertaking advanced oral health assessment** |
| Q19. Dental plaque |
| Q20. Dental calculus |
| Q21. Gum recession |
| Q22. Gum swelling |
| Q23. Tooth fracture (broken tooth) |
| Q24. Tooth wear |
