# Supplementary material for: Oral Healthcare Knowledge, Attitudes, Confidence and Learning Experiences Among Chinese Nursing Students: A Mixed-Methods Study
Source: Int Dent J. 2025 Nov 2;76(1):103994. doi: 10.1016/j.identj.2025.103994 (PMC12603743; doi:10.1016/j.identj.2025.103994)
Supplement: Supplementary file 2 [file mmc2.doc]

**Outline of the semi-structured interview**

1.Could you describe the primary channels through which you acquire knowledge about oral health? Among these, which do you consider most effective for learning, and why?

2.Please share your specific experiences in acquiring oral health knowledge and skills. Were there any particularly memorable moments or encounters? How did you feel at the time?

3.In your view, what methods or strategies might enhance the learning of oral health knowledge? Are there particular areas in which you hope to gain more in-depth understanding in the future?

4.From your perspective, what is the significance of nurses possessing essential knowledge and skills in oral health? Could you explain why?
